# Patent Ductus Arteriosus Stenting via Percutaneous Common Carotid Artery Access for Ductus Dependent Pulmonary Blood Flow in Newborns and Infants: Experience in Latvia

**DOI:** 10.15388/Amed.2024.31.2.5

**Published:** 2024-12-04

**Authors:** Zanda Grīnberga, Elizabete Zaharāne, Pauls Sīlis, Valts Ozoliņš, Normunds Sikora, Elīna Ligere

**Affiliations:** 1 Faculty of Residency, Riga Stradins University, Latvia; 2 Children’s Clinical University Hospital, Department of Paediatric Cardiology and Cardiac Surgery, Riga, Latvia; 3 Department of Paediatrics, Riga Stradins University, Latvia; 4 Department of Surgery, Riga Stradins University, Latvia

**Keywords:** PDA stenting, percutaneous transcarotidal approach, duct-dependent pulmonary circulation, cardiac catheterisation, pediatric cardiology, Raktažodžiai: PDA stentavimas, perkutaninis transkarotidinis metodas, nuo latako priklausoma plaučių kraujotaka, kardiokateterizavimas, vaikų kardiologija

## Abstract

**Background:**

Newborn patients with cyanotic congenital heart diseases with ductus-dependent pulmonary blood flow require neonatal repair, or palliation with a secure source of pulmonary blood flow, up to definitive surgical correction or palliation of the malformation. There is growing experience of percutaneous patent ductus arteriosus stenting to maintain the ductal flow. Patients in need of PDA stenting are newborns or small infants and recent data suggests that a weight of <4 kg increases the risk of thrombosis from femoral arterial catheterisation. Carotid access for newborn cardiac catheterisation avoids femoral arterial injury and improves the catheter course for certain transvasal procedures.

**The aim of this study:**

was to report the Riga Children’s Clinical University Hospital`s (CCUH) 7 year experience of patent ductus arteriosus stenting using the percutaneous transcarotidal approach in newborn babies and small infants with ductus-dependent critical heart diseases.

**Methods:**

A retrospective review of all newborn and small infants who underwent transcatheter arterial duct stenting through the percutaneous carotid artery approach at the CCUH in Riga, Latvia between the years 2013 and 2020.

**Results:**

In total, 8 patients underwent PDA stenting using the transcarotid approach between the years 2013 and 2020 in CCUH. The approach in all cases was chosen based on the anatomical features seen on echocardiography. In two cases, early restenting was necessary, while other patients had no procedure-associated complications. In the long term follow-up of 4 patients in two cases, dopplerography of the accessed common carotid artery showed stenotic changes up to 50%.

**Conclusions:**

PDA stenting using the transcarotid approach is currently considered a relatively safe method and does not have a greater risk of developing postprocedural complications compared to the transfemoral approach. Transcarotidal PDA stenting in neonates and small infants with ductus-dependent critical congenital heart disease is possible in small volume paediatric cardiac surgery centre to stabilise the patient prior to definitive surgery or palliation of complex CHD. The vascular access should be chosen depending on the anatomical features of the patient and the competency of the cardiac interventionalist. From our experience, long-term changes in the affected common carotid artery may develop in a substantial number of cases, they may not be clinically significant in midterm follow-up period but have to be reevaluated. However, further randomised studies are necessary with large cohorts and longer follow-up period.

## Abstract (it)

**Įvadas:**

Naujagimiams, sergantiems cianotinėmis įgimtomis širdies ligomis su nuo latakų priklausoma plaučių kraujotaka, reikalingas naujagimio atstatymas arba paliatyvioji pagalba, užtikrinant saugų plaučių kraujotakos šaltinį, iki galutinės chirurginės korekcijos arba paliatyvaus apsigimimo pašalinimo. Vis daugiau patirties įgyjama perkutaninio patento latakų arterijos stentavimo srityje, kad būtų palaikoma latako tėkmė. Pacientai, kuriems reikalingas PDA stentavimas, yra naujagimiai arba maži kūdikiai, o naujausi duomenys rodo, kad <4 kg svoris padidina trombozės riziką atliekant šlaunies arterijos kateterizaciją. Karotidinė prieiga atliekant naujagimio širdies kateterizaciją padeda išvengti šlaunies arterijos sužalojimo ir pagerina kateterio eigą atliekant tam tikras transvazalines procedūras.

Šio tyrimo tikslas – pateikti Rygos vaikų klinikinės universitetinės ligoninės (RVUL) 7 metų patirtį atliekant patento arterijos latakų stentavimą perkutaniniu transkarotidaliniu būdu naujagimiams ir mažiems kūdikiams, sergantiems nuo latakų priklausomomis kritinėmis širdies ligomis.

**Metodai:**

Retrospektyvinė visų naujagimių ir mažų kūdikių, kuriems 2013–2020 m. CCUH (Ryga, Latvija) buvo atliktas transkutaninis arterinio latakėlio stentavimas perkutaniniu miego arterijos metodu, apžvalga.

**Rezultatai:**

Iš viso nuo 2013 iki 2020 m. RVUL 8 pacientams buvo atliktas PDA stentavimas transkarotidiniu metodu. Visais atvejais metodas buvo pasirinktas atsižvelgiant į echokardiografijoje matomus anatominius požymius. Dviem atvejais prireikė ankstyvo restentavimo, o kitiems pacientams su procedūra susijusių komplikacijų nebuvo. Ilgalaikio stebėjimo metu 4 pacientams dviem atvejais, doplerografijos būdu atliktoje privestos bendrosios miego arterijos stenozės pokyčiai siekė iki 50 %.

**Išvados:**

Šiuo metu PDA stentavimas transkarotidiniu metodu laikomas gana saugiu metodu ir, palyginti su transfemoraliniu metodu, nesukelia didesnės komplikacijų po procedūros atsiradimo rizikos. Transkarotidinis PDA stentavimas naujagimiams ir mažiems kūdikiams, sergantiems nuo latakų priklausomomis kritinėmis įgimtomis širdies ydomis, galimas nedideliame vaikų kardiochirurgijos centre, siekiant stabilizuoti paciento būklę prieš galutinę operaciją ar sudėtingos KŠL paliatyvų gydymą. Kraujagyslinė prieiga turėtų būti parenkama atsižvelgiant į paciento anatomines savybes ir širdies intervencinės chirurgijos specialisto kompetenciją. Mūsų patirtis rodo, kad nemažai daliai atvejų gali atsirasti ilgalaikių pakitimų pažeistoje bendrojoje miego arterijoje, jie gali būti kliniškai nereikšmingi vidutinės trukmės stebėjimo laikotarpiu, tačiau turi būti vertinami iš naujo. Tačiau būtina atlikti tolesnius atsitiktinių imčių tyrimus su didelėmis grupėmis ir ilgesniu stebėjimo laikotarpiu.

## Introduction

Congenital heart defects (CHD) occur in ~ 1 in every 110 births, and ~ 25% of cases comprising a group known as critical congenital heart defects [1]. Newborns with cyanotic congenital heart diseases and ductus-dependent pulmonary blood flow require neonatal definitive surgery or palliation to ensure pulmonary blood flow until definitive surgical correction can be performed. There are three methods currently available: surgical systemic-to-pulmonary artery shunt – modified Blalock–Taussig shunt (mBTS), long-term use of prostaglandin E_1_ (PGE1), or minimally invasive percutaneous patent ductus arteriosus (PDA) stenting. PDA stenting was first described in 1991 in an animal model, creating the idea that it would be possible to develop this method for neonates in the future [2]. Nowadays, the creation of a mBTS is still a utilised method, but minimally invasive transcatheter methods are becoming more popular [3]. The most appropriate method is chosen based on the particular patient’s needs and anatomical features. Newborns or small infants are usually the patients who require PDA stenting and recent data suggests that a weight of < 4 kg increases the risk of thrombosis from femoral arterial catheterisation. Carotid access for newborn cardiac catheterisation avoids femoral arterial injury and improves the catheter course for certain transvasal procedures [4].

The aim of this study was to report the Riga Children’s Clinical University Hospital’s (CCUH) 7-year experience of patent ductus arteriosus stenting using the percutaneous transcarotidal approach in newborn babies and small infants.

## Materials and Methods

A retrospective review of all 8 patients (newborn and small infants) who underwent transcatheter arterial duct stenting through the percutaneous carotid artery approach at the CCUH in Riga, Latvia within the years 2013 to 2020 was carried out. The clinics for Paediatric Cardiology and Cardiac Surgery is the only facility in Latvia where children with CHD are treated and followed up. Our country has low population of 1.883 million inhabitants and birth rate during the years 2013–2020 was 20.346 ± 1641 life born per year [5]. The study was approved by the Committee of Ethics at Riga Stradins University.

## Statistical analysis

The data was compiled and analysed using MS Office Excel and IBM SPSS programs. Due to a very small sample size demographic and anthropometric data are provided as median, interquartile range, minimal and maximal values as well as absolute numbers.

## Procedure

All patients with congenital heart disease in Latvia undergo treatment and follow-up at the CCUH. The transcarotidal ductus stenting in our clinic was commenced on 2013. The same invasive cardiologist who is also a paediatric cardiac surgeon performed all the procedures there. Vital signs as heart rate, respiratory rate, blood pressure and oxygen saturation were monitored during procedures. The procedure was commenced with a surgical cut. The carotid artery was exposed and a single purse string suture was placed. The artery was then punctured and a guide sheath was placed. Aortography was performed to obtain the correct arterial duct size and localisation. The ductus arteriosus was catheterised and stented with the appropriate coronary artery stent. After placement of the stent, repeated aortography was done to check the position and flow through the stent. An additional stent was inserted in cases where migration of the stent was observed or if the stent did not completely cover the ductus arteriosus. An increase in oxygen saturation after the procedure was considered to be a good result for the procedure. If the stent position was accurate, the sheath was removed and the purse string suture was tied. The wound was closed and covered with a sterile bandage. The procedure was performed under systemic heparinisation with activated clotting time (ACT) monitored. After the procedure the infants were monitored in the Neonatal Intensive Care Unit (ICU). The patency of carotid artery used for the puncture in early postoperative period was checked during routine postprocedural echocardiography in the ICU, because specialised vascular ultrasound of the neck vessels was not available during the study period. The patients underwent repeated diagnostic or interventional angiographies with haemodynamic evaluation prior planning the next surgical intervention (Table 3). Follow up vascular dopplerography of the carotid arteries was performed during this follow-up study in medium/long term follow-up (follow-up period in Table 1).

**Table 1:** Patients demographical data, transcarotidal approach site, incidence of complications, follow-up period.

Median age (days) (IQR)	12.5 (6.5–31.5)
**Median weight (grams) (IQR)**	2715 (2567–3027)
**Gender: male/female**	5/3
**Carotidal approach: right/left**	0/8
**Guide sheath size: 3 Fr/4 Fr**	1/7
**Successful access, n (%)**	8 (100)
**Successful procedure, n (%)**	8 (100)
**Early postprocedural complications, n *(%)***	2 (25)
**Late postprocedural complications (associated with carotidal arteries, n (%)**	2 (25)
**Use of acetylsalicylic acid post procedure, n (%)**	8 (100)
**Median time at follow up for late complications (years) (IQR)**	7.5 (3.17–7.75)

**Table 2:** Diagnoses of the study patients, indications for transcarotidal approach, age and weight at the time of procedure, duration of the procedure, complications, dopplerography findings at the follow-up.

No.	Diagnosis	Birth weight (grams)	Diagnostic modality prior PDA stenting	Indications for transcarotidal approach	Age at PDA stenting (days)	Duration of procedure (min)	Early complications	Follow-up dopplerography, late complications	Follow-up time after PDA stenting
1.	TOF, PA	2230	Dg angio just before PDA stenting	Anatomical features – long and severely tortuous arterial duct	13	180	No	Both carotid arteries folded in an S shape, stenosis up to 50%. Indications for MRA	5 y 6 mo
2.	PA, HRHS. Coronary artery fistula	2960	Dg angio (age 8 days)	Anatomical features – arterial duct tortuous	15	240	No	No changes in carotid arteries, changes in vertebral arteries, indications for MRA	7 y 9 mo
3.	PA, HRHS	2640	Dg angio just before PDA stenting	Anatomical features – arterial duct tortuous	6	235	Restenosis – repeated stenting at 13 days of age	Left carotid artery distal 1/3 50% stenosis, indications for MRA	7 y 6 mo
4.	TOF, PA	2790	Dg angio just before PDA stenting	Anatomical features – long and severely tortuous arterial duct	12	115	No	Lost to follow up	8 y 4 mo
5.	Congenitally Corrected Transposition of the Great Arteries, PA, VSD, Dextrocardia	3970	Dg angio just before PDA stenting	Anatomical features – tortuous arterial duct	8	195	Restenosis – repeated stenting at 92 days of age	Was not performed	1 y 2 mo
6.	TOF, AVSD, PA	2140	Dg angio just before PDA stenting	Anatomical features –tortuous arterial duct	39	220	No	Was not performed	3 y 2 mo
7.	TOF, PA	3050	Dg angio (age 8 days)	mBT shunt thrombosis and also anatomical features – arterial duct tortuous	43	125	No	No changes	7 y 9 mo
8.	PA, Ventricular inversion, VSD, Double ductus arteriosus	2590	Dg angio (age 20 days)	Anatomical features – arterial duct tortuous	28	300	No	*Exitus letalis* at age of 45 days	*Exitus letalis* at age of 45 days

Abbreviations: AVSD – atrio-ventricular septal defect; Dg angio – diagnostic angiography; HRHS – hypoplastic right heart syndrome; mo – months; MRA – magnetic resonance angiography; PA – atresia of pulmonary valve; PDA – patent ductus arteriosus; TOF – Tetrology of Fallot; y – years; VSD – ventricular septal defect.

**Table 3:** Further angiographies, interventions and final surgical correction of the study patients.

No.	Diagnosis	Total number of angiographies	Age, Redilatation/restenting of PDA/RVOT stenting	Biventricular final anatomy 1-yes/2-no(univentricular)	Age, Surgical correction	Last follow-up time after PDA stenting
1.	TOF, PA	5	5 mo: hybrid operation, transventricular perforation of pulmonary artery valve, RVOT stenting, dilatation of LPA; 11 mo: transfemoral restenting of RVOT, redilatation of LPA; 1 y 4 mo: transfemoral restenting of RVOT, redilatation of LPA; 3y 2 mo: transfemoral dilatation and stenting of LPA.	1	1) 3 y 9 mo: longitudinal sternotomy, evacuation of RVOT stent, longitudinal dissection of stent in LPA**, dissection of PDA** (stent thrombosis), LPA plastics with xenoparicardium patch, VSD plastics with xenopericardial patch, RVOT infundibulectomy and reconstruction with Contegra conduit 16mm, atrial fenestration	5 y 6 mo
2.	PA, HRHS, Coronary artery fistula	4	6 mo: dg angio, stenting of mBT (5mm stent); 4y10 mo: Dg angio, occlusion of venous collateral.	2	30 days: longitudinal sternotomy, **stented PDA closure with titanium clip**, mB-T shunt (Goretex 4mm), atrial septectomy, TV closure 6 mo: resternotomy, closure of mBT shunt, bidirectional cavo-pulmonary anastomosis; 4 y. 10 mo: resternotomy, total cavo-pulmonary anastomosis (extracardiac tunnel Goretex conduit 18mm, fenestration 4mm).	7 y 9 mo
3.	PA, HRHS	5	14 days: repeated transcarotidal PDA stenting (same left transcarotidal approach, stent-in-stent); 3 mo: ASD stenting (not successful, embolization); 8 mo: Dg angio; 4 y 6 mo: Dg angio.	2	3 mo: sternotomy, atrial septectomy, evacuation of embolized stent; 8 mo: resternotomy**, PDA stent closure with titanium clip**, bidirectional cavo - pulmonary anastomosis (Glenn anastomosis), balloon dilatation of coarctation of aorta; 4 y 7 mo: resternotomy, total cavo-pulmonary anastomosis (extracardiac tunnel Goretex 18mm, fenestration 4mm).	7 y 6 mo
4.	TOF, PA	2	7 mo: transfemoral redilatation and restenting of PDA.	1	11 m: sternotomy, RVOT plastics with Goretex monocusp, **PDA stent closure with titanium clip**, VSD plastics with xwnopericardium patch	8 y 4 mo
5.	Congenitally Corrected Transposition of the Great Arteries, PA, VSD, Dextrocardia	5	3 mo: left transcarotidal PDA redilatation, restenting (stent 5mm diam); 1 y 5 mo: Dg angio; 2 y 2 mo: Dg angio; 2 y 2 mo: transvasal VSD occlusion.	1	2 y 2 mo: sternotomy: Sennings operation, Rastelli operation with Contegra 14mm, VSD plastics, **intraoperative PDA stent resection from RPA, plastics of LPA, RPA;** 2 y mo: resternotomy: plastics of VSD TV, evacuation of VSD occluder.	1 y 2 mo
6.	TOF, AVSD, PA	3	5 mo: transcarotidal PDA stent redilatation; 6 mo: transcarotidal RVOT restenting (6mm).	1	10 mo: longitudinal sternotomy, AVSD plastics with autopericardium and xenopericardium patch, evacuation of RVOT stent, RVOT and Pa plastics (autopericardium monocusp), Pa transannular xenopericardium patch, **plastics of LPA/resection of PDA stent**	3 y 2 mo
7.	TOF, PA	5	23 days: Dg angio (mBT thrombosis); 43 days: transcarotidal PDA stenting; 7 mo: Dg angio.	1	10 days: mBT shunt (Goretex 3mm); 1 y 4 mo: resternotomy, VSD plastics and RVOT infundibulectomy, plastics with xenopericardium, opening of native pulmonary valve, clipping ans dissection of stented PDA, clppping of mBT shunt.	7 y 9 mo
8.	PA.,Ventricular inversion, VSD, Double ductus arteriosus	2	*Exitus letalis* at age of 45 days (SVT, heart insufficiency)			

Abbreviations: AVSD – atrio-ventricular septal defect; ASD – atrial septal defect; Dg angio – diagnostic angiography; HRHS – hypoplastic right heart syndrome; LPA – left pulmonary artery; mBT – modified Blalock–Taussig shunt; mo – months; PA – atresia of pulmonary valve; Pa – pulmonary artery; PDA – patent ductus arteriosus; RPA – right pulmonary artery; RVOT – right ventricle outflow tract; TOF – Tetrology of Fallot; TV – tricuspid valve; y – years; VSD – ventricular septal defect.

## Results

Ductus arteriosus stenting using the carotidal artery approach was performed on 8 patients, 5 boys (63%) and 3 girls (37%) at the CCHU at median age 12.5 (6.5–31.5) days between 2013 and 2020. Patient demographic data, transcarotidal approach site, incidence of complications and follow-up is shown in Table 1. In our review of clinical cases, the most common congenital heart defect was tetralogy of Fallot with pulmonary atresia – in 4 patients, while the rest had other types of combined congenital heart disease, such as a congenitally corrected transposition of the great arteries with pulmonary atresia, pulmonary artery hypoplasia, etc. (Table 2). The diagnosis was detected antenatally in 50% of the patients.

In all but one case, the carotidal approach was chosen based on the anatomical features of ductus arteriosus observed through echocardiography. For example, if the origin from the underside of the aortic arch or the ductus was tortuous. We had only one case where this approach was used due to thrombosis in a previously created mBTS, the arterial duct had a distinctly tortuous course too. In all cases, the left common carotid artery was used for the procedure, the duration of the operation was 201 ± 62 minutes. All patients received an infusion with PGE_1_ prior to the procedure. A successful outcome from PDA stenting was defined as an increase in SpO_2_ and it was observed in all cases.

After the procedure, all patients received antithrombotic therapy with acetylsalicylic acid. Two patients developed postoperative complications in the early postoperative period: 1) unsatisfactory patency of stent and PGE1 restarted; 2) supraventricular paroxysmal tachycardia (SVT) episode. Both required restenting, while the other patients had no procedure-associated complications. All 7 surviving patients subsequently underwent the next stages of surgery.

Six of the study participants had long term follow-up by paediatric cardiologists and continue to be monitored regularly. Unfortunately, one of the children has been lost to follow-up. At the end of current follow-up period the examination of the dynamics of neck blood vessels by dopplerography was possible in four clinical cases. The common carotid showed stenotic changes with luminal narrowing of up to 50% in two of the cases, but no changes were detected in the other two. Magnetic resonance angiography is further recommended for all patients with changes.

The patients’ subsequent angiographies and operations are shown in Table 3. From the 7 survived patients 5 had biventricular correction of CHD, but 2 total cavo-pulmonary connection.

**Image 1:**
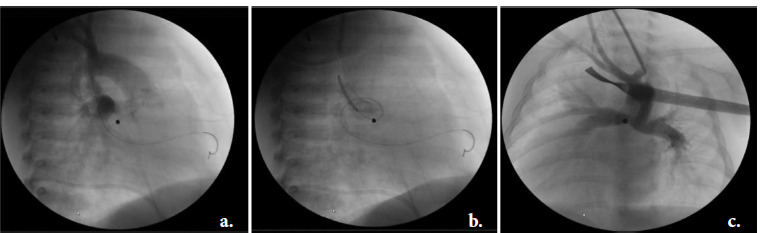
Angiography of transcarotidal PDA stenting (8 days old girl with congenitally corrected transposition of the great arteries, atresia of pulmonary valve, ventricular septal defect, dextrocardia: a) partially covered aortic end of arterial ductus (PDA); b) extra coronary stent inserted; c) pulmonary circulation via stented PDA (good result).

## Discussion

PDA stenting is an advanced palliative operation for patients with critical congenital heart disease who require secure PDA dependent pulmonary blood in the long term, before either full anatomic repair or second-stage of single ventricle palliation. Three methods are currently available: the creation of a systemic-to-pulmonary artery shunt (mBTS), the long-term use of PGE1, or percutaneous PDA stenting. The data from retrospective comparative studies reveals that PDA stenting is a good, less invasive alternative to surgical shunt creation. It is associated with earlier intervention, early improved hemodynamics, a reduced hospital stay and a better chance of survival. Despite the fact that it is possible to avoid potential postoperative complications with PDA stenting, e.g., recurrent laryngeal nerve injury, chylothorax, adhesions, etc., mBTS creation is still a method that plays a significant role in ensuring pulmonary blood flow in patients with PDA-dependent heart disease. Meta-analysis data from Tseng et al. (2022) reveals that mortality is similar for both methods despite the advantages of PDA stenting [6]. PDA stenting should not just be considered as an alternative to mBTS. Both methods are equally good and an appropriate method should be chosen for each patient individually [7, 8, 9, 10, 11].The data from previous more extensive studies suggests that a weight of < 4 kg increases the risk of thrombosis from femoral arterial catheterisation [4]. In the study by Glatz et al. (2018), the rate of acute arterial occlusion after femoral artery catheterisation in infants of < 4 kg was 20% to 30% (it was often clinically silent, correlated with the sheath size and inversely with patient weight) [15]. The neonatal carotid artery is larger in diameter than the femoral artery, therefore, transcarotid access may play a role in preventing femoral artery thrombosis in the high-risk small infant population [4]. Our patients were neonates or small infants (median age 12.5 (6.5–31.5) days, median weight 2.7kg) so were potentially high-risk group patients.

There are still discussions about the preferred access for PDA stenting, but it is known that the choice of access site is fundamental for a good procedural result. Traditionally, a transfemoral approach is chosen for paediatric cardiac intervention, but nowadays a transcarotid or transaxillary approach has been used more and more in specific cases. The transcarotid route is a relatively new approach for PDA stenting as it provides better access to the left side of the heart. Most concerns are related to its safety and potential complications – blood vessel damage or probable cerebrovascular events. However, current studies show that there is a low risk of vascular complications and there are good diagnostic imaging options for observing and noticing the changes in the carotid artery [12, 13].

According to the 2011 AHA scientific statement, cases with complex PDA anatomy with more tortuous ductus (> 2 turns) is a technical challenge for stenting and there is a great risk of procedural failure or the necessity for restenting. In these cases, the choice of vascular approach depends on institutional or surgeon expertise [14]. Currently, there are no unified guidelines for the best approach, but there are a few publications with other clinical experiences. All of them recommend the choice of access route based on PDA tortuosity and origin. In addition, from our experience, the carotid approach was mainly chosen due to anatomical features. For example, in their publication, Glatz et al. present a classification scheme for ductal morphology. They suggest the classification of PDAs into three groups depending on morphology and tortuosity (Tortuosity index). This classification would not only help in choosing appropriate vascular access, but could also allow to predict possible complications and the possibility of restenting [11,15].

The greatest concern when choosing an appropriate vascular approach is the potential for procedural and postprocedural complications. Possible complications from choosing a transcarotid approach are arterial thrombosis, ductal spasm and hematoma, which could cause respiratory problems, stenosis and subsequent neurological damage [16]. Despite the concerns, studies confirm that the approach is safe. In their retrospective study, Choudhry et al. (2015) reviewed infants under 3 months (n=18) who underwent catheterisation via the percutaneous carotid artery approach. In their study, access was achieved in all cases and minor complication occurred in only two cases – hypotension and ductal spasm [17]. Justino and Petit (2016) recommend a Doppler ultrasound performed within 24 hours post intervention to avoid early complications. In the long term, the carotid artery was classified postprocedurally as normal, mildly stenotic (lumen < 50% of the normal adjacent vessel), moderately stenotic (lumen reduced to > 50% of normal), or completely occluded [4]. Lahiri et al. (2021) analysed the effect of percutaneous carotid artery access on cerebral perfusion. They measured cerebral near infrared spectroscopy (NIRS) before, during and after the procedure in 48 patients with a median age of 23 days who underwent cardiac catheterisation via the common carotid artery. They discovered that this approach did not alter cerebral perfusion significantly [18]. Unfortunately, there are no extensive studies yet that show us the neurological outcome after the neonatal transcarotidal approach in the long term, into adulthood.

Regardless of the approach, PDA restenosis is a relatively frequent postprocedural complication and the anatomical and procedural risk factors are still being studied [3]. The Congenital Cardiac Research Collaborative published research data in 2021, where PDA restenting was necessary in 39% of all cases. Anatomical risk factors are not modifiable, as the most common ones were the mentioned PDA morphology, ductal tortuosity and single–ventricle anatomy. This could be explained by the fact that single-ventricle anatomy is also one of the most frequent indications for PDA stenting. The reason for restenting is not always stent dysfunction, but sometimes to extend the time to the second-stage procedure. Despite the probability of stenosis, it is usually noticed early and is not associated with other complications [8,15,16,19]. Extensive studies about postoperative complications at the site of puncture in carotidal artery have been published by Ligon et al. (2019). They also confirm transcarotid access as being a safe approach. In their study, the most common postprocedural complication was moderate stenosis – in approximately 35% of cases. One of the possible causes of restenosis could be the altered flow and neointimal proliferation [20]. In our study, 4 patients were monitored for long term complications from carotidal artery changes. Two of them developed moderate stenosis in the carotid artery without indications for intervention, but with a need for close follow-up.

## Limitations

The present study has a few limitations. Our study population included only 8 patients. This can be explained by the small overall population of Latvia and the rare incidence of these pathologies. Consequently, we also had limitations in the usage of statistical analysis. In this case, only descriptive statistical methods could be used, which did not allow us to make conclusions about the general population. Lastly, only 4 of the 6 patients (1 patient died, and 1 was lost to follow-up) underwent an appropriate assessment of long-term complications, such as dopplerography of the neck vessels.

## Conclusions

PDA stenting using the transcarotid approach is currently considered a relatively safe method and does not have a greater risk of developing postprocedural complications compared to the transfemoral approach. Transcarotidal PDA stenting in neonates and small infants with ductus dependent critical CHD is possible in small volume paediatric cardiac surgery centre to stabilise the patient prior to definitive surgery or palliation of complex CHD. The vascular access should be chosen depending on the anatomical features of the patient and the competency of the cardiac interventionalist. From our experience, long-term changes in the affected common carotid artery may develop in a substantial number of cases, may not be clinically significant in midterm follow-up period, but have to be reevaluated. However, further randomised studies are necessary with large cohorts and a longer follow-up period.
